# Clinical evaluation of sodium hypochlorite/amino acids and cross-linked hyaluronic acid adjunctive to non-surgical periodontal treatment: a randomized controlled clinical trial

**DOI:** 10.1007/s00784-023-05271-0

**Published:** 2023-09-23

**Authors:** Egle Ramanauskaite, Vita Machiulskiene, Yoshinori Shirakata, Urte Marija Dvyliene, Irena Nedzelskiene, Anton Sculean

**Affiliations:** 1 Clinic of Dental and Oral Pathology, Lithuanian University of Health Sciences, Eiveniu 2, 50103 Kaunas, Lithuania; 2 Department of Periodontology, Kagoshima University Graduate School of Medical and Dental Sciences, Kagoshima, Japan; 3 Department of Periodontology, University of Bern, Bern, Switzerland

**Keywords:** Periodontitis, Non-surgical periodontal therapy, Cross-linked hyaluronic acid, Sodium hypochlorite/amino acids

## Abstract

**Objectives:**

To compare the clinical outcomes obtained with either mechanical subgingival debridement in conjunction with a sodium hypochlorite and amino acids containing gel followed by subsequent application of a cross-linked hyaluronic acid gel (xHyA) gel, or with mechanical debridement alone.

**Materials and Methods:**

Fourty-eight patients diagnosed with stages II-III (Grades A/B) generalised periodontitis were randomly treated with either scaling and root planing (SRP) (control) or SRP plus adjunctive sodium hypochlorite/amino acid and xHyA gels (test). The primary outcome variable was reduction of probing depth (PD), while changes in clinical attachment level (CAL), bleeding on probing (BOP) and plaque index (PI) were secondary outcomes. The outcomes were assessed at baseline, at 3 and 6 months following therapy.

**Results:**

All patients completed the 6 months evaluation. At 6 months, the test group showed statistically significantly better results in terms of mean PD reduction (2.9 ± 0.4 vs 1.8 ± 0.6 mm, *p* < 0.001). Similarly, mean CAL gain was statistically higher in the test group compared to the control one (test: 2.6 ± 0.5 vs control: 1.6 ± 0.6 mm, *p* < 0.001). Mean BOP decreased from 81.8 ± 16.2% to 48.9 ± 14.5% in control (*p* < 0.001) and from 83.2 ± 15.5% to 17.6 ± 11.5% in test (*p* < 0.001) groups with a statistically significant difference favouring the test group (*p* < 0.001). Mean PI scores were reduced statistically significantly in both groups (from 38.8 ± 26% to 26.5 ± 20.5% in control (p = 0.039) and from 60.6 ± 10.9% to 12.7 ± 8.9% in test group (*p* < 0.001)), with a statistically significant difference between the groups (*p* < 0.001). The number of moderate pockets (4–6 mm) were reduced from 1518 (41.2%) to 803 (22.6%) in the control and from 1803 (48.6%) to 234 (7.7%) in the test group with a statistically significant difference between the groups (*p* < 0.001), while the number of deep pockets (≥ 7 mm) changed from 277 (7.6%) to 35 (1.0%) in the control and from 298 (8.7%) to 4 (0.1%) in test group (*p* = 0.003).

**Conclusion:**

Within their limits the present data indicate that: a) both treatments resulted in statistically significant improvements in all evaluated clinical parameters, and b) the adjunctive subgingival application of sodium hypochlorite/amino acid and xHyA to SRP yielded statistically significantly higher improvements compared to SRP alone.

**Clinical relevance:**

The combination of sodium hypochlorite/amino acid and xHyA gels to subgingival mechanical debridement appears to represent a valuable approach to additionally improve the outcomes of non-surgical periodontal treatment.

Clinical Trial Registration Number NCT04662216 (ClinicalTrials.gov).

## Introduction

Periodontitis is a chronic multifactorial inflammatory disease caused by dysbiotic dental plaque biofilms with the formation of an inflammatory infiltrate that contributes to destruction of connective tissue attachment to the tooth, alveolar bone resorption and may result in tooth loss [1–5]. In case of periodontitis a disruption of the normal function of the healthy subgingival plaque biofilm with concomitant disruption to its functional properties in relation to innate defense surveillance and tissue maintenance, leading to excessive, deregulated inflammation and tissue destruction is observed [6, 7].

Primary clinical features of periodontitis include the loss of periodontal tissue support, which manifests through clinical attachment loss and radiographically assessed alveolar bone loss with the presence of gingival bleeding and periodontal pockets [5]. The recently published clinical practice guidelines for treating stage I–III periodontitis concluded that cause-related therapy is aimed at reducing/eliminating the subgingival biofilm and calculus by means of subgingival instrumentation, which may include the adjunctive application of physical or chemical agents [8].

Recent systematic reviews have provided some evidence indicating that adjunctive aids, in conjunction with mechanical debridement, might enhance the outcomes of non-surgical periodontal therapy [9–12]. More recently, findings from *in vitro* experiments have shown, that a sodium hypochlorite gel has a softening effect on the extracellular biofilm matrix which in turn, may facilitate its mechanical removal. It has been shown that the effect of sodium hypochlorite/amino acid gel is due to its active part, the chloramine, which forms following the chlorine transfer of sodium hypochlorite to the amine functions of the added amino acids [13]. Amino acids act like a buffer and provide protection to soft tissues. The high pH (11) of this formulation has a softening effect on the calculus, which makes the cleaning process easier [14]. Therefore, it may be anticipated that during subgingival debridement treatment, both the mechanical and chemical components act synergistically to disrupt the hard and soft biofilm which in turn, may facilitate granulation tissue removal [13, 14]. In this respect, positive clinical effects of a sodium hypochlorite gel were reported in studies treating residual periodontal pockets [15, 16], peri-implant mucositis [17] and peri-implantitis [14].

HA is a naturally occurring biodegradable polymer that is responsible for several structural properties of tissues as a component of the extracellular matrix [18]. Several studies have provided evidence indicating that HA plays an important role in wound healing, supports scarless wound-healing, promotes angiogenesis and has a bacteriostatic effect in surgical wounds [19–22]. When used during periodontal surgery, HA has been shown to promote periodontal regeneration in intrabony, recession and furcation defects [23–25]. Clinical studies revealed that HA may represent a valuable constituent to mechanical debridement (i.e., scaling and root planing), thus resulting in statistically significant clinical improvements, evidenced by reduction in probing depth (PD), gain of clinical attachment (CAL) and improved bleeding on probing (BOP) values, compared to scaling and root planing alone [26–29].

Recently, a novel concept consisting of enhancing biofilm removal during nonsurgical therapy by means of a sodium hypochlorite/amino acids followed by application of a cross-linked hyaluronic acid gel (xHyA) gel was suggested as a novel strategy to improve the outcomes of nonsurgical periodontal therapy [30, 31]. Results from two case series have shown statistically significant clinical improvements compared to baseline following scaling and root planing in conjunction with sodium hypochlorite/amino acid and xHyA, thus suggesting that this strategy may represent a valuable novel strategy in non-surgical periodontal treatment.

However, to the best of our knowledge, at present no randomized controlled clinical trials have evaluated the potential clinical relevance of this novel concept as compared to mechanical debridement alone.

Therefore, the aim of this randomized controlled clinical study was to compare the clinical outcomes obtained with either mechanical subgingival debridement in conjunction with sodium hypochlorite/amino acid gel followed by subsequent application of xHyA, or with mechanical debridement alone.

## Material and methods

### Study design

This study was conducted as a 6-months prospective, examiner-blind, randomized controlled clinical trial with a parallel design. The study was performed according to CONSORT guidelines for randomized controlled clinical trials (http://www.consort-statement.org/) [32]. Ethical permission was issued by the Regional Biomedical Research Ethics Committee (No. BE-2–87). Prior to participation, all patients signed a written informed consent form. After signing the informed consent form, the patients were randomly assigned to the control or test groups (allocation ratio 1:1). The study was conducted between September 2019 and January 2022. In addition, the study protocol was registered at ClinicalTrials.gov, NCT04662216.

### Study population

All patients included in the study were enrolled and treated at the Department of Dental and Oral Pathology at the Lithuanian University of Health Sciences in Kaunas, Lithuania.

Inclusion criteria:

- Males and females ≥ 18 years old.
- Periodontitis stages II–III, grades A/B, generalised [5].
- Good general health (i.e., absence of systemic diseases and no intake of medication which may affect periodontal health).
- Presence of at least 20 teeth (wisdom teeth excluded).
- Absence of removable dentures.
- Patients willing to provide written informed consent and willing to complete the 6-month study follow-up.

Exclusion criteria:

- Patients already included in other clinical trials.
- Smokers.
- Periodontal treatment during the last 12 months.
- Antibiotic treatment 3 months prior to the start of the trial.
- Antibiotic prophylaxis required for dental treatment.
- Ongoing medication that may affect the clinical features of periodontitis.
- Pregnant/lactating.
- Allergies to sodium hypochlorite

### Sample size calculation

At the start of the study, a significance level of α = 0.05, a relevant average difference in PD of 1 mm between study groups with a standard deviation of 1 mm and a power (1—α) of at least 0.8 were set to calculate the minimum number of necessary cases (at least 20 per group). Assuming any possible dropouts during the study period, the number of patients was increased to 24 in each group. A power calculation at the end of the study with the given number of cases yielded a power of 99.6%.

### Periodontal treatment

Baseline periodontal measurements were obtained 2 weeks prior to the treatment, which was followed by professional supragingival tooth cleaning and individual oral hygiene instructions for all included patients. These treatments included manual toothbrushes and interdental brushes. All patients were provided the same type of toothpaste (Elmex Enamel Protection, Gaba GmbH, Germany) and tooth (CS 5460, Curaprox, Curaden, Switzerland) and interdental (TePe, Tepe Mundhygienprodukten, Sweden) brushes. Oral hygiene instructions were reinforced at each follow-up visit, but no further treatment was rendered.

Two weeks later, under local anaesthesia, subjects in the control group underwent full-mouth SRP performed with ultrasonic (Satelec/Acteon suprasson newtron ultrasonic scaler) and hand instruments (LM SharpDiamond 1/2, 7/8, 11/12, 13/14 SD mini Gracey and Gracey curettes, LM Dental™, Finland). Subsequently, all teeth were polished using a low-abrasive paste (Lunos Super Soft, RDA < 5, Dürr Dental, Germany). Mechanical debridement took on average 3.5 h per patient.

**Fig. 1:**
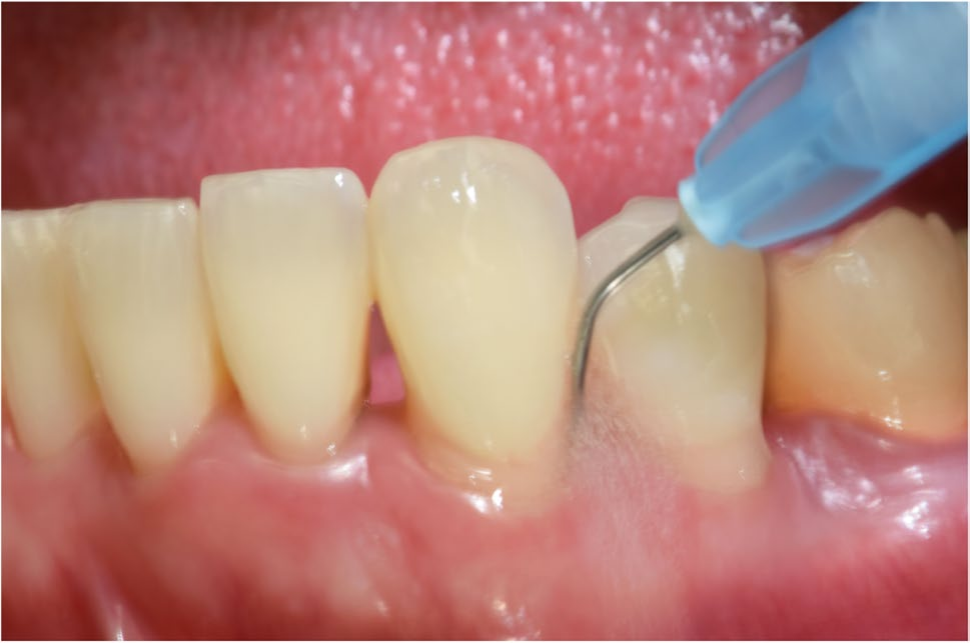
Application of sodium hypochlorite/amino acid gel to the periodontal pocket

**Fig. 2:**
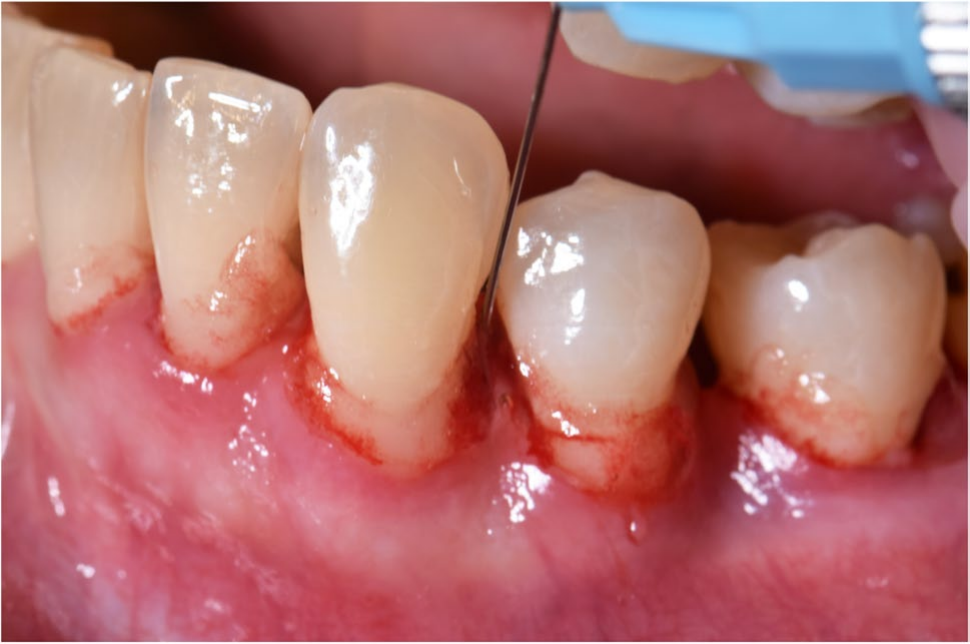
Application of a mixture of natural and cross-linked hyaluronic acid (high molecular)to the periodontal pocket

In the test group, full-mouth SRP was performed as follows: in all pockets with PD ≥ 4 mm a sodium hypochlorite/amino acid gel (Perisolv®, Regedent AG, Zürich, Switzerland) was instilled into the pockets and kept there for 60 s before subgingival instrumentation. Subgingival instrumentation was carried out with the same ultrasonic and hand instruments and the application of sodium hypochlorite/amino acid gel was repeated until the instrumentation was considered sufficient (i.e., for a total of 2–3 times) (Fig. 1). All treatments were performed with magnifying glasses (4.5X – Ergo Advanced, Univet, Rezzato BS, Italy) and sufficient instrumentation was attained when root surfaces exhibited smooth surfaces upon probing with an explorer probe (Explorer-Periodontal Probe 8-520B, LM Dental™, Finland). Following SRP, a mixture of natural and cross-linked hyaluronic acid (high molecular) gel (Hyadent® BG, Regedent AG, Zürich, Switzerland) was instilled in the pockets using a blunt needle (Fig. 2).

### Clinical measurements

The following clinical parameters were assessed using a Williams periodontal probe to the nearest mm (LM 51 ES, LM-Dental™, Finland) on all teeth at 6 sites per tooth (i.e., mesio-buccal (mb), mid-buccal (b), disto-buccal (db), mesio-oral (mo), mid-oral (o) and disto-oral (do)) at baseline (T0), 3 months (T1) and 6 months (T2) following the treatment:

- Bleeding on probing (BOP), defined as the percentage of sites positive to bleeding within 10 s after probing (%). BOP was assessed for treated sites (PD ≥ 4 mm) and full mouth (FMBOP).
- Plaque index (PI), defined as the percentage of sites with visual plaque on the tooth surface (%). PI was assessed at treated sites (PD ≥ 4 mm) as well as the full mouth (FMPI).
- Probing depth (PD), measured in millimetres from the gingival margin to the bottom of the probed pocket.
- Recession (REC), measured in millimetres from the gingival margin to the cemento-enamel junction or to the margin of a cervical restoration.
- Clinical attachment level (CAL), calculated by adding PD and REC at each site.

At each visit, the clinical examiner had to record possible complications or adverse events related to the tested materials or study interventions, as well as those reported by study subjects.

### Outcomes

For data analysis, PDs were subdivided into two categories: moderate (PD 4–6 mm) and deep (PD ≥ 7 mm). The primary outcome variable was the mean PD change from baseline to 6 months in moderate sites. Secondary outcome variables included PD change in deep pockets at 6 months, as well as CAL changes in moderate and deep sites. In addition, mean BOP and PI changes from baseline to 6 months in all treated sites (PD ≥ 4 mm) and the full mouth were evaluated.

### Blinding

Clinical measurements and initial supragingival tooth cleaning were performed by a blinded calibrated examiner (U.M.D.), who was not aware in any of the cases of the type of treatment performed. All recordings were made without access to previous measurements to avoid bias.

To ensure blindness, the treatment procedures were performed by one experienced periodontist (E.R.).

The patients were not aware to which group they had been assigned. Periodontal treatment was performed in a sterile field (face drapes were used) to eliminate the possibility for patients to observe the procedure.

A third investigator (I.N.), unaware of the type of treatment performed, processed coded data for statistical analysis.

### Randomization and allocation concealment

Forty-eight patients were randomized into two treatment groups. A computer-generated randomization table was created. Patients were assigned unique numbers from 1 to 48, and 2 sets of randomized numbers were generated (24 for control group subjects and 24 for test). Allocation concealment was performed using sealed envelopes to be opened before periodontal treatment. The generation of the random sequence allocation and the assignment of participants to interventions were performed by the investigator, distinct from the clinical examiner and the clinician who performed the treatment.

### Calibration

Five patients, not related to the study, each diagnosed with periodontitis stages II–III [5], were used to calibrate the examiner (U.M.D.). The examiner was asked to evaluate PD, REC, CAL, BOP and PI at 6 sites per tooth on 2 separate appointments, 48 h apart. Calibration was accepted if measurements at baseline and at 48 h were equal to the millimetre at > 90% level. The examiner was not aware of the procedure to be performed.

### Statistical analysis

Statistical analysis was performed with the IBM SPSS 27 software package (IBM Corp.). Data analysis was performed using the patient as the statistical unit. For all clinical parameters, mean values per subject and per visit were calculated. In particular, PD and CAL of moderate pockets at baseline and at 3- and 6-month follow-ups were obtained by averaging PDs and CALs in moderate sites for each patient at baseline, 3- and 6-month follow-ups. Similarly, per-patient PD and CAL of deep pockets at baseline (and at 3 and 6 months) were obtained by averaging PD and CAL values in deep sites for each patient at baseline, 3 and 6 months. Per-patient BOP and PI were obtained by calculating a percentage share of tooth sites with BOP and plaque for each patient by classifying pockets by baseline PD (all treated sites with PD ≥ 4 mm and the full mouth).

The Shapiro–Wilk test was performed to assess whether clinical periodontal measures followed a normal distribution. If data followed a normal distribution, a paired-samples *t* test was performed to evaluate before- and after-treatment comparisons within groups. If the data did not follow a normal distribution, the Wilcoxon signed rank test was performed on related samples to assess before- and after-treatment comparisons within the groups. The between-group comparisons of measures were obtained by either the independent-samples *t* test (if a parameter followed a normal distribution) or the Mann–Whitney test (if a specific measure followed a non-normal distribution). The significance level was set at 0.05.

## Results

### Participant flow

**Fig. 3:**
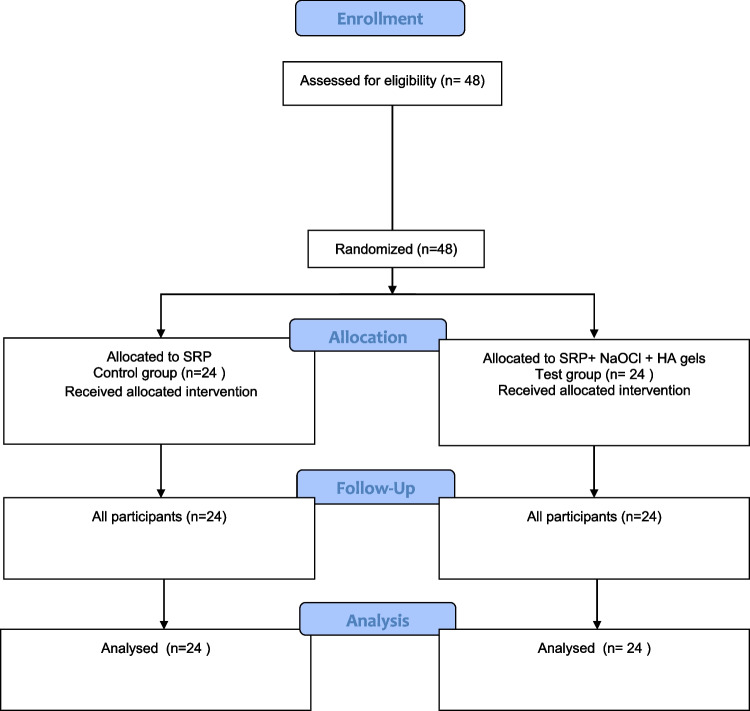
CONSORT flow diagram of participant recruitment

All 48 patients completed the study. Each treatment group (SRP or SRP + sodium hypochlorite/amino acid + xHyA) consisted of 24 randomly selected patients. A flowchart of the study is depicted in the CONSORT flow diagram (Fig. 3). In all subjects, healing was uneventful. No adverse effects of sodium hypochlorite/amino acid and xHyA were observed during the study period.

### Baseline characteristics

Clinical and demographic baseline characteristics of the 48 participants are shown in Table 1. The baseline examination revealed that the two study groups showed similar characteristics for PD, CAL, bleeding (BOP and FMBOP) and plaque scores with no significant differences between the groups (except for PI and FMPI) (Table 1A). Furthermore, regarding the number of type of treated teeth, no statistically significant differences were observed between control and test groups (Table 1B).

**Table 1:** Clinical and demographic characteristics of sample population at the baseline

A. Characteristics of sample population at the baseline							
		SRP (*N* = 24)		SRP + NaOCl + HA (*N* = 24)		*P* value	
Age (years)		49.3 ± 11.2		47.3 ± 10.7		0.53^a^, n.s	
Gender, *n* (%)							
Males		7 (29.2)		6 (25)		0.745^b^,	
Females		17 (70.8)		18 (75)		n.s	
Periodontitis stage, *n* (%)							
Stage II		16 (66.7)		17 (70.8)		0.134^b^,	
Stage III		8 (33.3)		7 (29.2)		n.s	
Grade A		13 (54.2)		12 (50.0)			
Grade B		11 (45.8)		12 (50.0)		0.242^b^, n.s	
PD (mm)		5.3 ± 0.6		5.2 ± 0.4		0.592^c^, n.s	
CAL (mm)		5.5 ± 0.5		5.6 ± 0.6		0.546^c^, n.s	
PI (%)		38.8 ± 26		60.6 ± 10.9		**0.002**^**c**^	
BOP (%)		81.8 ± 16.2		83.2 ± 15.5		0.687^c^, n.s	
FMPI (%)		35.7 ± 23.7		52.9 ± 11.4		**0.003**^**c**^	
FMBOP (%)		68.9 ± 20.3		76.5 ± 18.2		0.184^c^, n.s	
B. Distribution of treated teeth							
Treatment	Second Molars	First Molars	Second Premolars	First Premolars	Canines	Lateral Incisors	Central incisors
Control group (*n*)	88	84	89	91	94	94	95
Test group (*n*)	86	90	86	89	96	96	96
*p*	0.549	0.187	0.505	0.682	0.153	0.153	0.317

BOP – bleeding on probing; CAL – clinical attachment level; FMBOP – full-mouth bleeding on probing; FMPI – full-mouth plaque index; PD – probing depth; PI – plaque index

n.s. not significant

^a^ Independent-samples *t* test

^b^ Fisher’s exact test for the 2 × 2 table, sex by group (SRP, SRP + NaOCl + xHyA)

^c^ Mann–Whitney U test for two independent groups

Mann–Whitney U test for two independent groups

### Effect on clinical parameters

PD changes during the study period were analysed for different pocket categories: mean moderate (4–6 mm) and mean deep (PD ≥ 7 mm) pockets. Data is presented in Table 2.

**Table 2:** PD (mean (SD)) at sites with moderate (4–6 mm) and deep (≥ 7 mm) pockets

	Control group (*n* = 24)	Test group (*n* = 24)	*p* value
Moderate pockets (4–6 mm)			
Baseline After 3 months Baseline vs. 3 months After 6 months Baseline vs. 6 months	4.8(0.2) 2.9(0.7) < 0.001^b^ 3.0(0.6) < 0.001^b^	4.7(0.2) 2.2(0.4) < 0.001^b^ 1.8(0.4) < 0.001^b^	0.417^a^ < 0.001^a^ < 0.001^a^
Deep pockets (≥ 7 mm)			
Baseline After 3 months Baseline vs. 3 months After 6 months Baseline vs. 6 months	8.0(0.7) 4.4(1.4) < 0.001^b^ 4.3(1.0) < 0.001^b^	8.2(0.9) 2.9(1.1) < 0.001^b^ 2.4(1.0) < 0.001^b^	0.443^a^ < 0.001 ^a^ < 0.001

^a^ Statistical analysis by Student's t test for two independent groups

^b^ Paired Samples T Test for two dependent groups

**Fig. 4:**
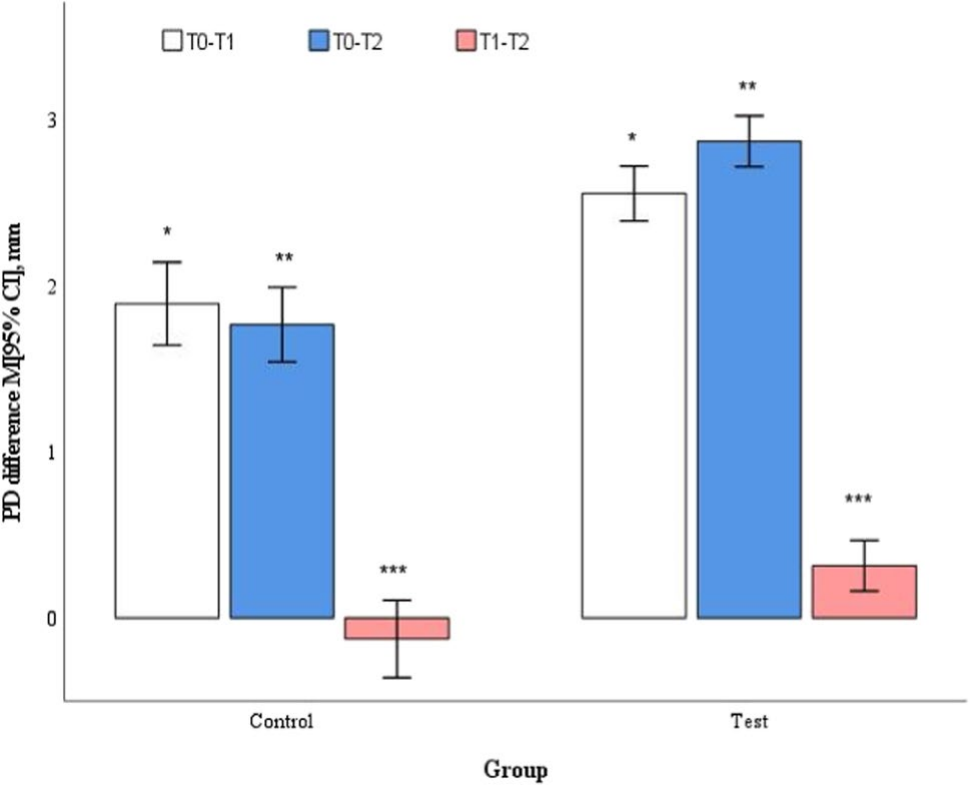
Mean changes in PD in moderate pockets (4-6 mm) at different time points. ^*,**^p < 0.001, ^***^p = 0.002 by Student's t test for two independent groups. T0 – baseline; T1 – 3 months follow-up; T2 – 6 months follow-up

In mean moderate pockets, the baseline values did not reveal a statistically significant difference between control and test groups (4.8 ± 0.2 and 4.7 ± 0.2, respectively, *p* = 0.417). Both groups showed statistically significant improvements at 3 and 6 months compared to baseline (*p* < 0.001); however, statistically significantly higher reductions were observed in favour for the test group at both points in time (*p* < 0.001) (Table 2). The change of PD between 3 and 6 months differed statistically significantly between groups in favour for the test group (*p* = 0.002) (Fig. 4).

**Fig. 5:**
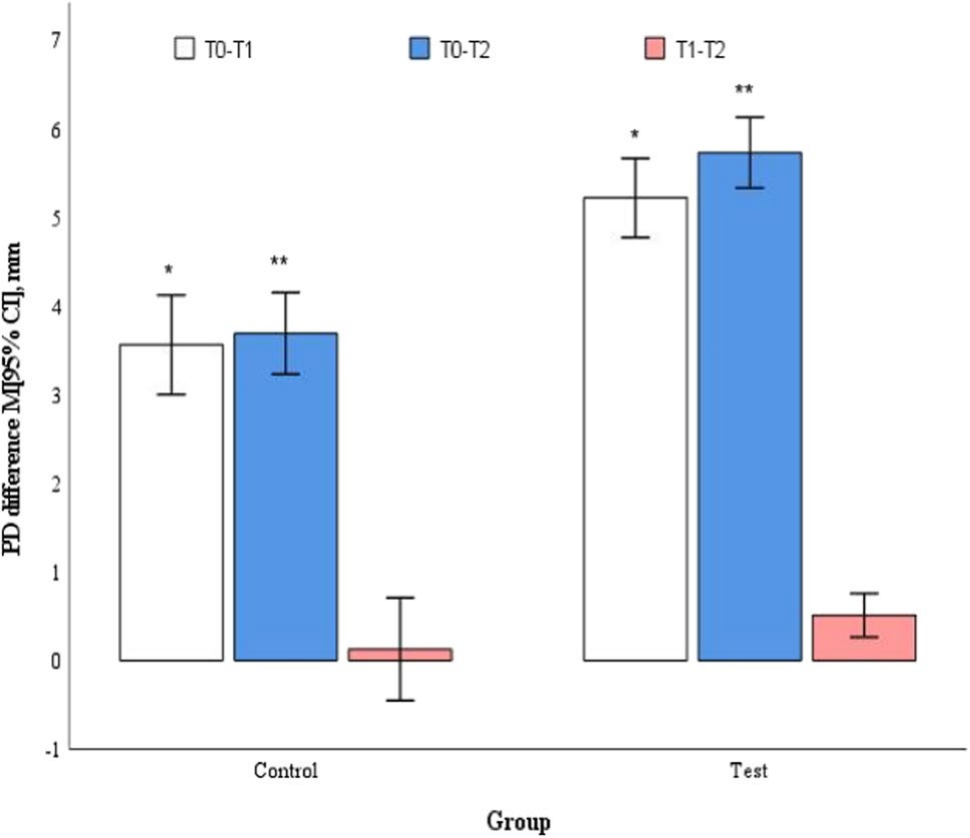
Mean changes in PD in deep pockets (≥ 7 mm) at different study time points. ^*,**^p < 0.001, by Student's t test for two independent groups. T0 – baseline; T1 – 3 months follow-up; T2 – 6 months follow-up

Baseline PD values in mean deep pockets category were not statistically significantly different between control and test groups (8.0 ± 0.7 and 8.2 ± 0.9, respectively, *p* = 0.443). Both groups reached statistically significant improvements at 3 and 6 months compared to baseline (*p* < 0.001); however, PD reduction in the test group was statistically significantly higher compared to the control group at both follow-ups (*p* < 0.001) (Table 2). The change between 3 and 6 months did not differ between the groups (*p* = 0.096) (Fig. 5).

CAL changes in mean moderate and mean deep pockets are reported in Table 3.

**Table 3:** CAL (mean ± SD) at sites with moderate (4–6 mm) and deep (≥ 7 mm) pockets

	Control group (*n* = 24)	Test group (*n* = 24)	*p* value
Moderate pockets (4–6 mm)			
Baseline After 3 months Base vs. 3 months After 6 months Base vs. 6 months	4.8(0.3) 3.1(0.8) < 0.001^b^ 3.1(0.7) < 0.001^b^	4.6(0.2) 2.4(0.6) < 0.001^b^ 2.0(0.5) < 0.001^b^	0.026^a^ < 0.001^a^ < 0.001^a^
Deep pockets (≥ 7 mm)			
Baseline After 3 months Base vs. 3 months After 6 months Base vs. 6 months	7.9(0.6) 4.5(1.2) < 0.001^b^ 4.6(1.0) < 0.001^b^	8.1(0.7) 3.2(1.4) < 0.001^b^ 2.8(1.3) < 0.001^b^	0.412^a^ 0.002 ^a^ < 0.001 ^a^

^a^ Statistical analysis by Student's t test for two independent groups

^b^ Paired Samples T Test for two dependent groups

**Fig. 6:**
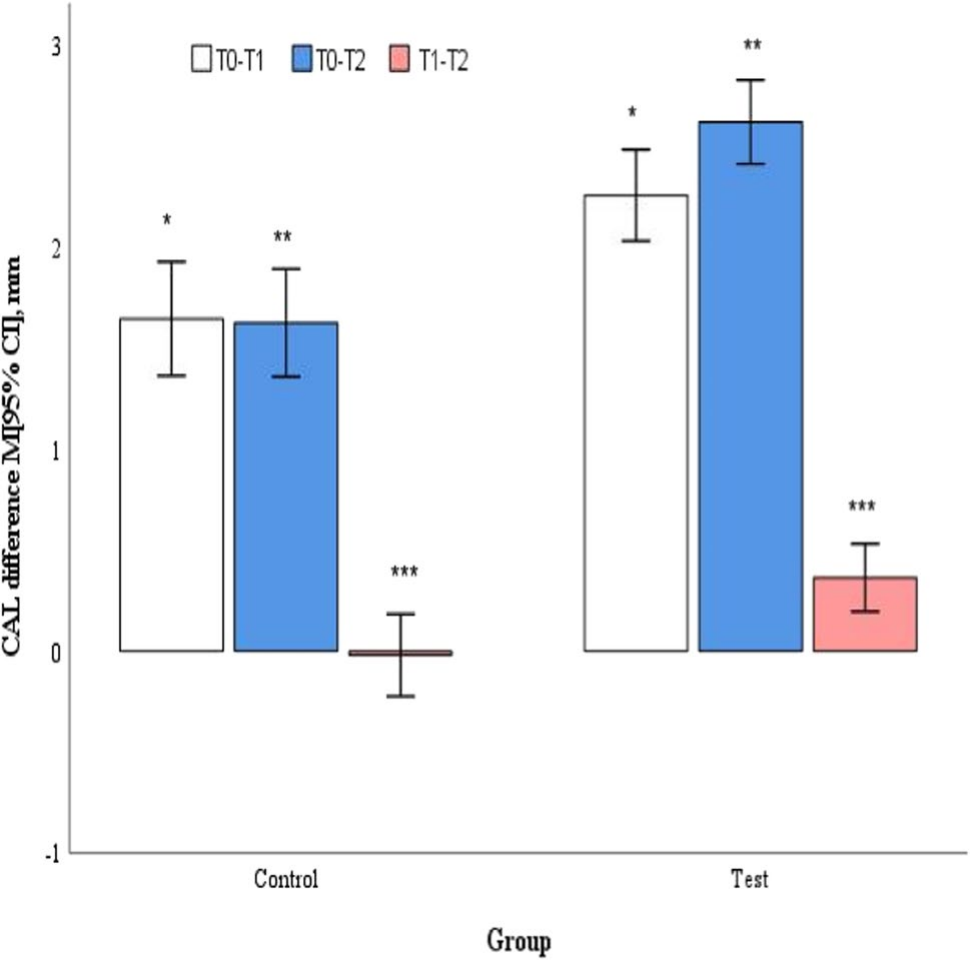
Mean changes in CAL in moderate pockets (4-6 mm) at different study time points. ^*,**^p < 0.001, ^***^p = 0.004 by Student's t test for two independent groups. T0 – baseline; T1 – 3 months follow-up; T2 – 6 months follow-up

At baseline, in mean moderate pockets group, the CAL values were slightly higher in the control group (4.8 ± 0.3 mm) compared to the test group (4.6 ± 0.2 mm; *p* = 0.026). Both groups reached significant improvements at 3 and 6 months compared to baseline (*p* < 0.001); however, a statistically significant difference between groups was observed in favour of the test group at both points in time (*p* < 0.001) (Table 3). Mean CAL change between the 3- and 6-month follow-ups was statistically significantly different between the groups in favour for the test group (*p* = 0.004) (Fig. 6).

**Fig. 7:**
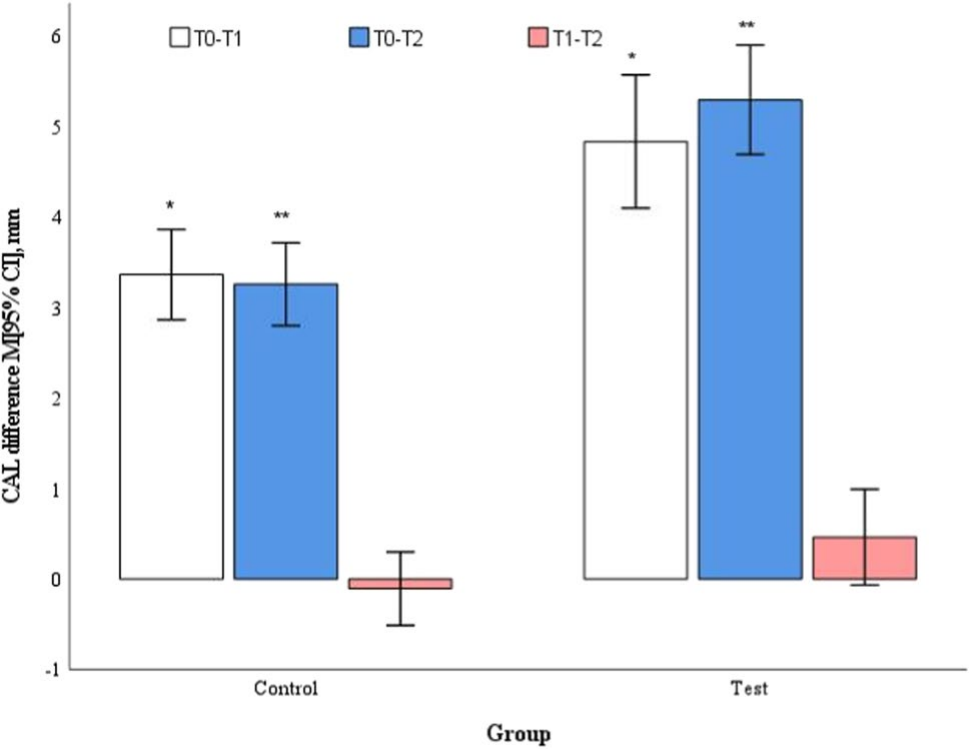
Changes in CAL in deep pockets (≥ 7 mm) at different study time points. ^*^p = 0.002, ^**^p < 0.001, by Student's t test for two independent groups. T0 – baseline; T1 – 3 months follow-up; T2 – 6 months follow-up

In mean deep pockets baseline, CAL values were not statistically significantly different and measured 7.9 ± 0.6 mm in the control group and 8.1 ± 0.7 mm in the test group (*p* = 0.412), respectively. Both groups reached statistically significant improvements at both follow-ups, compared to baseline (*p* < 0.001); however, statistically significantly better improvements were achieved in favour for the test group (*p* < 0.001) (Table 3). Mean CAL change between 3- and 6-month follow-up did not show a statistically significant difference between the groups (*p* = 0.077) (Fig. 7).

BOP changes were evaluated for treated sites (PD ≥ 4 mm) and full mouth (FMBOP).

Regarding full-mouth measurements, baseline FMBOP values were similar in test (76.5 ± 18.2%) and control (68.9 ± 20.3%) groups (*p* = 0.184). Both study groups reached significant improvements at 3 and 6 months compared to baseline (*p* < 0.001). The difference between groups was not statistically significant at the 3-month follow-up (*p* = 0.06) but reached a statistically significant difference in favour for the test group at the 6-month follow-up (*p* < 0.001) (Table 4).

**Table 4:** BOP (%) at treated sites (PD ≥ 4 mm) and full mouth (mean ± SD)

	BOP		*P* value	FMBOP		*P* value
	Control (*n* = 24)	Test (*n* = 24)		Control (*n* = 24)	Test (*n* = 24)	
Baseline	81.8 ± 16.2	83.2 ± 15.5	0.687^a^	68.9 ± 20.3	76.5 ± 18.2	0.184^a^
After 3 months	39.1 ± 15.9	28.3 ± 14.6	**0.018**^**a**^	33.3 ± 13.7	25.9 ± 12.3	0.06^a^
Baseline vs 3 months	** < 0.001**^**b**^	** < 0.001**^**b**^		** < 0.001**^**b**^	** < 0.001**^**b**^	
After 6 months	48.9 ± 14.5	17.6 ± 11.5	** < 0.001**^**a**^	40.8 ± 13.8	15.6 ± 9.9	** < 0.001**^**a**^
Baseline vs 6 months	** < 0.001**^**b**^	** < 0.001**^**b**^		** < 0.001**^**b**^	** < 0.001**^**b**^	

^a^ Statistical analysis by Student's t or Mann–Whitney test for two independent groups

^b^ Wilcoxon Signed Ranks Test for two dependent groups

The analysis of treated pockets (PDs ≥ 4 mm) revealed no statistically significant difference in baseline BOP values between test and control groups (*p* = 0.687). Although both groups showed statistically significant improvements at 3- and 6-month follow-ups compared to baseline (*p* < 0.001), the reduction of BOP was statistically significantly better in the test group compared to the control group at both points in time (*p* = 0.018 and *p* < 0.001, respectively) (Table 4).

PI changes were evaluated for treated sites (PD ≥ 4 mm) and the full mouth (FMPI).

Baseline FMPI values were higher in the test group (52.9 ± 11.4%) than in the control one (35.7 ± 23.7%) (*p* = 0.003). However, both groups showed significant improvements at 3- and 6-month follow-ups compared to baseline (*p* < 0.001). The intergroup comparison revealed a statistically significant difference between groups in favour for the test group at 6 months (*p* = 0.006) (Table 5).

**Table 5:** PI (%) at treated sites (PD ≥ 4 mm) and full mouth (mean ± SD)

	PI		*P* value	FMPI		*P* value
	Control	Test		Control	Test	
Baseline	38.8 ± 26	60.6 ± 10.9	**0.002**^**a**^	35.7 ± 23.7	52.9 ± 11.4	**0.003**^**a**^
After 3 months	20.3 ± 16.7	18.8 ± 11.4	0.714^a^	19.3 ± 15.0	17.1 ± 9.7	0.893^a^
Baseline vs 3 months	** < 0.001**^**b**^	** < 0.001**^**b**^		** < 0.001**^**b**^	** < 0.001**^**b**^	
After 6 months	26.5 ± 20.5	12.7 ± 8.9	**0.018**^**a**^	23.5 ± 16.6	11.2 ± 7.9	**0.006**^**a**^
Baseline vs 6 months	**0.039**^**b**^	** < 0.001**^**b**^		** < 0.001**^**b**^	** < 0.001**^**b**^	

^a^ Statistical analysis by Student's t or Mann–Whitney test for two independent groups

^b^ Wilcoxon Signed Ranks Test for two dependent groups

A similar pattern was observed in the analysis for PI at treated pockets. In particular, higher PI (%) values were reported in the test group than the control group (*p* = 0.002). Both study groups showed statistically significant improvements at 3- and 6-month evaluations, compared to baseline (*p* < 0.001). No statistically significant difference was observed between groups at the 3-month evaluation (*p* = 0.714), whereas at the 6-month examination, the reduction in PI was statistically significantly greater in the test group (*p* = 0.018) (Table 5).

### Analysis of frequency distributions of shallow, medium, and deep pockets

Additionally, the analysis of frequency distribution of shallow (1–3 mm), medium (4–6 mm) and deep (≥ 7 mm) sites at baseline, 3 and at 6 months was performed (Table 6). At baseline, subjects in the control group had 1518 (41.2%) sites with moderate pockets (4-6 mm) and test group 1803 (48.6%) sites, respectively. At 6 months this number reduced to 803 (22.6%) in control and 234 (7.7%) sites in the test group with a statistically significant difference between the groups (*p* < 0.001). Similarly, the number of deep pockets (≥ 7 mm) changed from 277 (7.6%) to 35 (1.0%) in control and from 298 (8.7%) to 4 (0.1%) in test at 6 months evaluation with a statistically significant difference between the groups (*p* = 0.003) (Table 6).

**Table 6:** Number of sites with shallow (1–3 mm), medium (4–6 mm) and deep (≥ 7 mm) pockets in test and control groups at different study timepoints

	1–3 mm			4–6 mm			≥ 7 mm		
	Control	Test	*P* value	Control	Test	*P* value	Control	Test	*P* value
Baseline	1916 (51.2%)	1603 (42.7%)	0.05*	1518 (41.2%)	1803 (48.6%)	0.041^*^	277 (7.6%)	298 (8.7%)	0.52
After 3 months	2938 (78.6%)	3284 (88.2%)	0.013*	728 (20.3%)	402 (11.5%)	0.018^*^	39 (1.1%)	12 (0.3%)	0.053
After 6 months	2859 (76.4%)	3398 (92.2%)	0.006^*^	803 (22.6%)	234 (7.7%)	< 0.001^*^	35 (1.0%)	4 (0.1%)	0.003*

Data in bold represents statistically significant differences between test and control groups

No sub-analysis between different tooth types was performed since the results are presented only for moderate (PD 4–6 mm) and deep sites (PD ≥ 7 mm) without including furcation involved teeth.

## Discussion

The present randomized clinical trial has investigated the clinical outcomes obtained with the subgingival application of a combination of sodium hypochlorite/amino acid and xHyA gels in conjunction with non-surgical periodontal therapy in untreated periodontitis patients. The results have shown that in patients diagnosed with stages II–III periodontitis, SRP combined with sodium hypochlorite/amino acid and xHyA gels resulted in statistically significantly higher clinical improvements evidenced through PD reduction, CAL gain, and decrease of BOP and PI values as compared to SRP alone.

An interesting observation of the study is related to PD and CAL changes between 3 and 6 months in moderate pockets. In particular, no statistically significant change was observed in the control group between the 3- and 6-month follow-ups, whereas in the test group, the change reached statistical significance. This observation appears to indicate that the test group demonstrated gradual improvements from month 3 to month 6, even though no additional treatment was performed. This finding may bear clinical relevance since it may suggest that the clinical improvements following the adjunctive sodium hypochlorite/amino acid and xHyA to SRP occur over a longer period of time (e.g., up to 6 months). Additionally, this observation may also suggest that a period of 3 months following nonsurgical periodontal therapy might be too early for making a final decision on the need for additional therapy (e.g., periodontal surgery). A similar pattern supporting the gradual improvement, was also observed for FMBOP and FMPI, where no statistically significant differences were observed between the groups at the 3-month follow-up, while it reached statistical significance at 6 months in favour of the test group.

This observation might be explained by the mode of action of xHA. In particular, the high molecular weight cross-linked HA that was used in this clinical trial can maintain its stability for 4 to 6 weeks which in turn, may serve as explanation for its prolonged activity [33].

When interpreting the clinical outcomes, it must be emphasized that the goal of non-surgical periodontal treatment is PD ≤ 4 mm with negative BOP [34]. The results of the current study have shown that the need for further treatment appears to be smaller in the test group, as demonstrated by the analysis of the change of number of moderate (4–6 mm) and deep pockets (≥ 7 mm) over time. In detail, in the control group, the total number of pockets with PD 4–6 mm decreased from 1518 to 803 with the corresponding values of 1803 and 234 in the test group. Similarly, the number of deep sites reduced from 277 to 35 in control and from 298 to 4 in test group.

As stated by Salvi et al., generally, a PD reduction of approximately 1–1.5 mm in moderate pockets (4–6 mm) and 2–2.5 mm in deep pockets (≥ 6 mm) can be expected [35] following mechanical debridement. This occurs concomitantly with CAL gain of approximately 0.5 mm in moderate pockets at baseline and 1.5 mm in deeper sites [35]. Any additional pocket reduction or CAL gain would, therefore, represent a true clinical benefit of the adjunctive materials used. This observation was also confirmed in the present study where in moderately deep sites, the mean PD change from baseline to 6 months measured 1.7 mm in the control group and 2.9 mm in test group, respectively, with the corresponding values of 3.7 mm and 5.8 mm, at deep sites (PD ≥ 7 mm). In moderately deep pockets, the mean CAL gain from baseline to 6 months measured 1.6 mm in the control group and 2.6 mm in test group, while in deep pockets, the corresponding values measured 3.2 mm and 5.3 mm, respectively.

When interpreting the results, one may ask the question to what extent each of the used adjunctive materials contributed to the additional improvements observed in the test group. In this respect, it is important to emphasize that the present study has used the combination of the two materials as a single concept, thus combining the effects of sodium hypochlorite/ amino acid gel to facilitate mechanical debridement and biofilm removal with the well-known wound-healing facilitating effects of xHyA. Based on previous findings from *in vitro* and animal experiments, it was hypothesized that the inherent effect of NaOCl to facilitate mechanical debridement and biofilm removal, may lend additional support to xHyA to express its wound healing improving properties [20, 23–25].

Despite the inherent positive effects of the used combination approach, it should be kept in mind that combining two materials and their use in conjunction with scaling and root planing also means a higher therapy effort in terms of time and costs. Additionally, it should be also emphasized that the present has only evaluated the outcomes in moderate (PD 4–6 mm) and deep sites (PD ≥ 7 mm) at teeth without furcation involvement. Obviously, further studies are warranted to evaluate the potential effect of this treatment approach in furcation involved teeth.

However, to the best of our knowledge, this is the first RCT evaluating the outcomes following the adjunctive application of sodium hypochlorite/amino acid gel and xHyA to scaling and root planing for untreated periodontal disease.

A recently published retrospective analysis of 29 clinical cases evaluated the adjunctive application of sodium hypochlorite/amino acid and a mixture of natural and cross-linked hyaluronic acid (high molecular) gels to SRP for treating residual periodontal pockets in patients diagnosed with periodontitis stages II–IV who were included into periodontal maintenance [30]. The authors reported an overall PD reduction exceeding 2 mm, associated with a similar CAL gain (2.02 mm). The results are comparable with the results obtained in this study. However, it must be emphasized that the study included compliant patients who already underwent nonsurgical periodontal treatment, as well as patients diagnosed with periodontitis stage IV, and therefore, direct comparisons are difficult. However, the same protocol has been evaluated in a very recent case series consisting of a total of twenty-one systemically healthy, non-smoking patients diagnosed with stage II-III periodontitis [31]. Compared to baseline, a statistically significant mean reduction of PD values was obtained after 3- and 6- months, amounting 2.6 ± 0.4 mm, and 2.9 ± 0.4 mm, respectively (*p* < 0.001), while mean CAL gain measured 2.3 ± 0.5 mm at 3- months, and 2.6 ± 0.5 mm at 6-months in comparison to baseline (*p* < 0.001). Mean reduction of BOP values amounted to 54.9 ± 16.9% at 3- months, and to 65.6 ± 16.4% at 6- months, respectively (*p* < 0.001). The number of moderate pockets (4–5 mm) reduced from 1808 at baseline to 274 at 6 months evaluation, and the number of deep (≥ 6 mm) pockets changed from 319 to 3, respectively [31]. These results compare well to those obtained in the present study, thus pointing to the potential clinical relevance of this novel clinical protocol.

Moreover, the adjunctive application of sodium hypochlorite/amino acid and hyaluronic acid gels to SRP has been tested separately in several clinical studies. On one hand, a recent clinical trial has evaluated the effect of the adjunctive application of sodium hypochlorite gel to SRP in residual periodontal pockets [9]. The findings revealed statistically significant PD reduction favouring the used of the sodium hypochlorite/amino acid gel, compared to a placebo (*p* = 0.028), as well as a statistically significant CAL gain at 6 months in the NaOCl-treated group, compared to the application of CHX gel (*p* = 0.0026).

One the other hand, the results of the studies on the adjunctive application of hyaluronic acid to non-surgical periodontal therapy are inconsistent. For instance, some of the studies found statistically significant improvements for the adjunctive application of hyaluronic acid to SRP in terms of PD and BOP reductions and CAL gain [27, 29], whereas in other studies adjunctive application of hyaluronic acid did not reach statistically significant differences in the investigated clinical parameters compared to SRP alone [36, 37].

Obviously, when interpreting the current results, certain the following limitations need to be mentioned: a) the study included a relatively small sample size and was of relatively short duration (i.e., 6 months), and b) only systemically healthy, non-smoking patients diagnosed with periodontitis stages II and III exhibiting adequate oral hygiene skills were included in the study.

## Conclusion

Within their limits the present data indicate that: a) Both treatments resulted in statistically significant improvements in all evaluated clinical parameters, and b) The adjunctive subgingival application of sodium hypochlorite/amino acid gel and xHyA to SRP yielded statistically significantly higher improvements compared to SRP alone.

## Data Availability

All data underlying the results are available as part of the article and no additional source data is applicable.
